# Extreme Heat Events and Emergency Department Visits among Older Adults in California from 2012–2019

**DOI:** 10.3390/medicina60101593

**Published:** 2024-09-28

**Authors:** Melodie Santodomingo, Edward M. Castillo, Lara Schwarz, Jesse J. Brennan, Tarik Benmarhnia, Theodore C. Chan

**Affiliations:** 1 Department of Emergency Medicine, School of Medicine, University of California, San Diego, CA 92093, USA; msantodomingo@health.ucsd.edu (M.S.); emcastillo@ucsd.edu (E.M.C.); jjbrennan@ucsd.edu (J.J.B.); 2 Scripps Institute of Oceanography, University of California, San Diego, CA 92093, USA; lnschwar@ucsd.edu (L.S.); tbenmarhnia@ucsd.edu (T.B.); 3 Herbert Wertheim School of Public Health and Longevity Science, University of California, San Diego, CA 92093, USA

**Keywords:** climate change, heat wave, geriatric, emergency department, healthcare utilization

## Abstract

*Background and Objectives*: Extreme heat events are increasing with climate change impacting human health. This study investigates the impact of extreme heat events on Emergency Department (ED) utilization by older adult patients. *Materials and Methods*: We conducted a study of all 324 non-federal hospital EDs in California during an 8-year period from data extracted from the California Department of Health Care Access and Information (HCAI). The study utilized a time-stratified case-crossover design to investigate ED visited in patients aged 65 years and older during 1-day and 2-day heat wave events. Extreme heat temperatures were measured and weighted using historical data at the zip code level at the 95th, 97.5th, and 99th percentiles 2012 through 2019. Conditional logistical regression was used to estimate the odds of ED visits during extreme heat events compared to non-extreme heat days. Stratified analyses by age and comorbidity status were conducted. *Results*: During the study period, 8,744,001 of ED visits among older patients were included in the study analysis. Odds ratios (OR) increased for during 1-day heat events (95th percentile (OR = 1.023, 95%CI: 1.020, 1.027), 97.5th percentile (OR = 1.030, 95%CI: 1.025, 1.035), 99th percentile (OR = 1.039, 95%CI: 1.032, 1.058)) and more so with 2-day heat wave events (95th percentile (OR = 1.031, 95%CI: 1.026, 1.036), 97.5th percentile (OR = 1.039, 95%CI: 1.031, 1.046), 99th percentile (OR = 1.044, 95%CI: 1.032, 1.058)). Older patients with three or more comorbidities had the highest odds of ED visits (OR = 1.085, 95%CI: 1.068, 1.112) at the 99th percentile. *Conclusions*: Our findings indicate that ED visits increase for older patients during extreme heat events, particularly with event intensity and duration. Older patients with at least one comorbidity were at greater risk.

## 1. Introduction

Emergency departments (EDs) in the U.S. and throughout the world will be challenged in the near future both by climate change and demographic change with aging populations [1]. Moreover, epidemiologic research has revealed an association between ambient temperatures and increased all-cause mortality among older adults where an increase of 1 degree Celsius produces up to a 5% increase in all-cause mortality [2]. Since the 2015 Paris Agreement to address global warming, worldwide temperature averages have already increased by 1.2 degrees Celsius, underscoring the urgency of comprehending and addressing the role of climate change on the health of humans, particularly older patients, and the overall impact on our healthcare systems [3].

Advancing age naturally impairs the body’s thermoregulatory mechanisms and its ability to maintain a homeostatic temperature range and respond to external and internal stimuli [4]. When combined with a heightened incidence of comorbidities in this population, this population is especially vulnerable to experiencing negative health outcomes during extreme heat events. This risk likely varies by geography and demographics of this population, and this burden is disproportionately shouldered by older individuals, who face unique health disparities exacerbated by the impact of extreme climate health events [5,6].

To better understand the scope of these challenges, we examine the impact of climate change on ED use by older adults in California. Due to its large size, unique and varied topography and climate, the state has experienced an increase in climate-related warming events including extreme heat events, prolonged droughts, wildfires, and other natural disasters. These events and their impact on human health and behaviors have resulted in public health challenges and health emergencies throughout the region [7,8,9,10].

The aim of this study was to investigate the role of extreme heat events on the health and healthcare utilization of older adults. Specifically, we sought to assess the impact of extreme heat events on the rate of ED visits by this population and what patient and environmental factors may play a role in ED utilization during these events.

## 2. Materials and Methods

### 2.1. Study Design

We conducted a large review of all ED visits in the state of California during an 8-year period. All 324 non-federal hospital EDs (excluding military and Veteran’s Administration hospitals) in the state were included in the study. Therefore, these data are representative of nearly all ED encounters in the state. Data were extracted from the California Department of Health Care Access and Information (HCAI) database and used to analyze ED visits during the months in which extreme heat events were most common (May through September) from 2012 to 2019. Data extracted included patient demographic data, co-morbidity and diagnoses, and other health-related variables. A detailed description of these data sources has been previously reported [11].

A time-stratified case-crossover design was applied to understand the effects of extreme heat on ED visits on the elderly population in California [12,13]. Cases were matched to a set of controls utilizing the same day of the week within the same month to compare the odds of an ED encounter during a heat event. This type of ecological study design was used to capitalize on the day-to-day variation in exposure levels and ED visits while adjusting for any potential time-fixed confounding, such as regional climate variability, by design [14].

Gridded observed data from the PRISM Climate Group of Oregon State University were used to estimate the daily temperature at the zip code level in California from 2012 to 2019 [15]. Extreme heat events were defined as any day within the historical temperature data for the summer period that exceeded the 95th, 97.5th, and 99th percentiles for 1 day or 2 consecutive days during the summer period from May to September, similar to previous studies in defining and analyzing heat events [16]. These definitions were applied to maximum temperature distributions to consider daytime and nighttime extreme heat events.

### 2.2. Statistical Analysis

Descriptive statistics were performed to understand the study population. Older adults were grouped into three separate categories as follows: 65–74, 75–84, and 85+. Sex, race/ethnic group, and ED disposition are reported. To robustly assess comorbidity status, we quantified the burden of morbidity using the Charlson Comorbidity Index (CCI) which include 17 categories of concurrent medical conditions—such as heart disease, diabetes, cancer, AIDS, etc.—that predict mortality [17]. CCI scores were grouped as an overall score of 0, 1, 2, or ≥3 where the higher the score, the greater the burden of comorbidity. A conditional logistic regression was then applied to the cases and matched controls days to evaluate the effect of extreme heat on ED visits in the elderly population overall. Stratified analyses were conducted by CCI score to explore the potential role of comorbidities in increasing vulnerability and ED presentation to extreme heat events for this population. The study protocol was reviewed and approved by our institutional human subjects review committee.

## 3. Results

Between 2012 and 2019, there were a total of 8,744,001 ED visits by older adults 65 years of age and older in California from May–September of those years. During the study period, the proportion of older adults presenting to EDs grew by 4% with a steady increase annually. Females in this age group presented to the ED more frequently than females. Overall, among adults who presented to the ED, approximately one-third (33.8%) were admitted. Table 1 shows the demographic, race/ethnicity, disposition, and CCI for ED visits by this patient population.

Table 2 highlights the six extreme heatwave categories used in this study similar to previously reported analyses [16]. These six categories were determined by the duration of the event, i.e., 1-day and 2-day durations, and temperatures that exceeded the 95th, 97.5th, and 99th thresholds based on historical climate data. Extreme heat events were generally well distributed across the study period and varied spatially across California [16].

**Figure 1:**
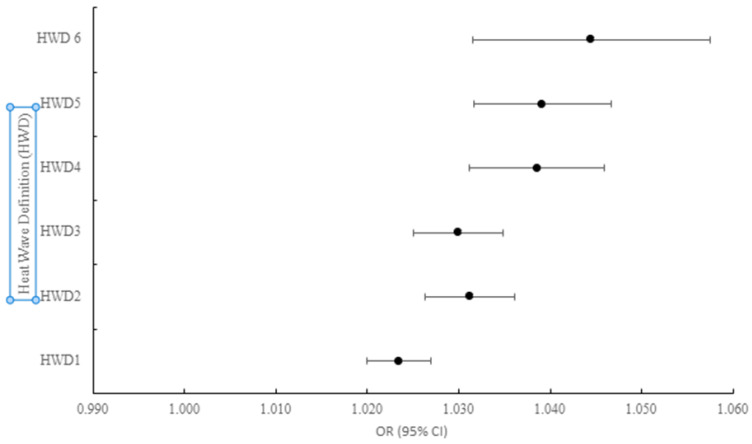
Odds ratios (ORs) and 95% confidence intervals (CIs) of heat wave impacts on emergency department visits among older adults in California, May–September 2012–2019.

Overall, older adults were more likely to present to the ED when heat wave temperatures were at or above the 95th percentile (Figure 1). There was a significant difference between 1-day heat wave events at the 95th percentile (OR:1.023, 95% CI: 1.02, 1.027) and 2-day heat wave events at the 99th percentile (OR: 1.044 95% CI: 1.032, 1.058) indicating temperature intensity and duration as vulnerability factors in this population. Overall, older adult patients were more likely to present to the ED with increasing temperature intensity and duration during extreme heat events.

When stratified by the Charlson Comorbidity Index (CCI) score, older patients with no comorbidities had lower rates of ED visits than those with a CCI score of 1 or more with multiple comorbidities showing a strengthened likelihood of ED presentation in older adults (Table 3). There was also a consistent positive effect for patients with each heat wave definition indicating that an increase in temperature, duration, or both increased the likelihood of ED presentation in this population.

## 4. Discussion

This study provides evidence that older adults have increased the odds of seeking ED care during extreme heat events. As this study represents all non-federal ED encounters in the state it provides an overview of how heat waves can impact healthcare utilization in the state. While the ORs might appear minimal, given the size of the encounters studied (over 8 million), the impact on total actual ED encounters is likely large. Therefore, these findings represent a potentially significant increase in ED encounters in the state.

This finding has significant healthcare and public health implications given the likelihood of increasing heat waves in the future as a result of climate change, as well as the aging population worldwide [18]. Previous work on ED visits and climate-sensitive exposures have focused on heat-related emergency conditions such as heat exhaustion and heat stroke [19]. Our study included visits for these conditions, as well as any other conditions that led to patients presenting to the ED for care.

Extreme heat events have detrimental effects on the health of older individuals [20]. The aging process compromises the body’s ability to regulate temperature, making older adults more susceptible to heat-related illnesses such as heat stroke, dehydration, and exacerbation of underlying chronic conditions [7]. The increased ED usage during these extreme weather events highlights the vulnerability of older adults and the strain placed on healthcare resources and public health infrastructure.

There are several factors that may contribute to increased ED usage among older adults during heat waves. Limited access to air conditioning, especially for those living alone or in low-income settings, can drive older individuals to seek emergency care as a means of seeking relief from the extreme heat [16]. Social isolation further compounds the problem, as older adults may lack support systems to help them cope with the challenges posed by heat waves [21]. A recent study indicated that older patients are more likely to miss primary care appointments during extreme temperature events [22]. Additionally, inadequate public health messaging and awareness among older adults about heat-related health risks may result in the delayed recognition and management of heat-related illnesses, leading to ED presentations as a last resort [20].

The surge in ED visits during heat waves poses challenges in terms of resource allocation and healthcare delivery [3]. Crowding in EDs not only leads to longer wait times and decreased quality of care but also diverts resources from other critical patient needs. These challenges are particularly concerning in the context of an aging population, where the demand for healthcare services is already high [23].

The results of this study add to the literature by identifying not only that this population is at risk but that those with co-morbidities, such as cardiovascular, respiratory, and metabolic disease, and higher CCI scores are more vulnerable and more likely to present to the ED for care during heat events [2,4,6,7]. As extreme heat events and climate-related natural disasters are becoming predictable, future work should expand on this understanding.

By using a range of different metrics to define heatwave events and patient-level demographics information, this study also highlights environmental climate event factors that put this population at risk. Both duration and heat intensity were shown to increase the odds of older adults presenting to the ED for care, respite, and relief. Using thresholds based on past climate data to define extreme heat events is useful in providing an evidence-based structure for activating interventions in vulnerable populations during extreme heat events based on intensity and duration and may be effective in decreasing serious health-related illnesses [10].

Extreme heat events have emerged as a significant public health concern, exacerbated by the ongoing climate crisis [24]. As temperatures soar, the impact on older adults becomes increasingly pronounced, leading to an upsurge in ED utilization placing a strain on healthcare resources. It is important that public health measures prioritize older adults in heat action plans as these events become more frequent in the coming years. Although there have been public health interventions like cooling centers aimed at preventing negative health outcomes from extreme heat events, this population will likely require multi-faceted public health interventions to prevent avoidable ED visits [6,9,25].

Potential interventions and solutions would include advanced monitoring systems and cooling support structures pinpointing specific microclimate areas at particular risk on a regional level at a given time. Others include community-wide efforts to reduce risks during heat events such as adjusting the time of activities including sporting and other events to avoid peak temperatures during heat events. Finally, individual measures such as wearable sensors that monitor and alert individuals who may be reaching a point of high risk may be of utility. By implementing comprehensive public health interventions, it may be possible to mitigate the adverse effects of heat waves on older adults and enhance resilience during heat waves.

### Limitations

While this was a large study conducted with hundreds of EDs over multiple years, this study is limited by its retrospective data collection. The analysis correlated multiple databases including hospital and ED data with climate databases stratified by region. However, roughly 3% of zip codes reported did not successfully match to the temperature database, thus impacting ED data for those regions and time periods. In addition, while we utilized a case-crossover design, this methodology may be limited especially given the significant regional heat wave event variability by time and location.

Moreover, this study was conducted in California, the largest state in the U.S. by population. California has a unique, diverse, and varied topography with coastal regions, valleys, mountain ranges, arid deserts and other climatic zones, where the impact of heat waves and extreme heat events may differ significantly by region even within a single county [26]. In addition, while 55% of California’s landmass is considered rural with vast and varied terrain, the state is highly urbanized where 94% of its population is considered to live in urban areas [27]. In urban settings, where older adults tend to relocate, increased temperature is concentrated with a potential “heat island effect”, reporting a 1–7-degree Fahrenheit increase in ambient temperatures compared to its surrounding areas [28,29]. Moreover, general demographic differences between urban and rural areas may play a role in access to emergency care. Rural areas generally lack the same access to transportation, infrastructure, and social support due to resource allocation and other sociodemographic factors such as income which may be confounding variables in our study [27].

Finally, our study focused on extreme heat events defined as 1- or 2-day events above the 95th percentile for historical climate data by calendar in the months of May through September when these events commonly occur. This study did not account for those events that occurred in fall and winter months, rare as they may be. In addition, the impact of climate change on human health in an increasingly warm environment will likely extend well beyond just those days of extreme heat events [30].

## 5. Conclusions

In this multi-year study throughout California, older adults presented more frequently to the ED during extreme heat events for medical care. Patient co-morbid conditions as well as environmental factors, such as the duration and intensity of the heat event, increased ED utilization in this population. In light of climate change and the aging population, these findings may have significant policy and public health implications, including providing guidance for public health and preparedness planners to address the needs of this population during such extreme heat events.

## Figures and Tables

**Table 1:** Descriptive statistics (total *n* = 8,744,001).

Age	*n*	%
65–74	3,835,490	43.9
75–84	2,918,799	33.4
85+	1,989,712	22.8
Sex *		
Female	4,994,571	57.1
Male	3,749,191	42.9
Race/Ethnicity		
Hispanic	1,813,809	20.7
Non-Hispanic White	5,050,370	57.8
Non-Hispanic Black	657,753	7.5
Non-Hispanic American Indian/Alaska Native	30,364	0.3
Non-Hispanic Asian/Pacific Islander	783,500	9.0
Unknown	408,205	4.7
ED Disposition		
Admission	2,955,043	33.8
Discharge	5,487,920	62.8
SNF/ICF	142,439	1.6
AMA	98,624	1.1
Expired	28,577	0.3
Other	31,398	0.4
CCI		
0	3,429,488	39.2
1	1,993,940	22.8
2	1,141,707	13.1
>=3	2,178,866	24.9
Year		
2012	929,000	10.6
2013	952,113	10.9
2014	1,011,339	11.6
2015	1,063,212	12.2
2016	1,118,655	12.8
2017	1,183,463	13.5
2018	1,211,379	13.9
2019	1,274,840	14.6

* Missing *n* = 239.

**Table 2:** Heat wave definitions.

Heat Wave Definition (HWD)	Percentile	Duration, Days
HWD1	95	1
HWD2	95	2
HWD3	97.5	1
HWD4	97.5	2
HWD5	99	1
HWD 6	99	2

**Table 3:** Odds ratios (ORs) and 95% confidence intervals (CIs) of heat wave impacts on emergency department visits among older adults by Charlson Comorbidity Index (CCI) in California, May–September 2012–2019.

	CCI = 0	CC1 = 1	CCI = 2	CCI ≥ 3
Heat Wave Definition (HWD)	OR (95% CI)	OR (95% CI)	OR (95% CI)	OR (95% CI)
HWD1	1.01 (1.005, 1.016)	1.020 (1.013, 1.027)	1.030 (1.020, 1.040)	1.044 (1.037, 1.052)
HWD2	1.01 (1.008, 1.023)	1.029 (1.019, 1.040)	1.034 (1.021, 1.048)	1.057 (1.047, 1.067)
HWD3	1.02 (1.009, 1.024)	1.024 (1.013, 1.034)	1.040 (1.027, 1.054)	1.053 (1.043, 1.063)
HWD4	1.02 (1.007, 1.030)	1.036 (1.021, 1.052)	1.044 (1.023, 1.065)	1.071 (1.055, 1.086)
HWD5	1.03 (1.015, 1.038)	1.030 (1.015, 1.046)	1.040 (1.019, 1.061)	1.067 (1.052, 1.083)
HWD6	1.03 (1.007, 1.048)	1.028 (1.002, 1.055)	1.048 (1.012, 1.085)	1.085 (1.058, 1.112)

## Data Availability

Data for this study were obtained from publicly archived datasets including the California Department of Health Care Access Information (https://hcai.ca.gov/healthcare-utilization/emergency-department (URL accessed on 27 September 2024)) and the PRISM Climate Group (https://prism.oregonstate.edu/historical/ (URL accessed on 27 September 2024)).
